# Analyzing the Impact of Various Jump Load Intensities on Countermovement Jump Metrics: A Comparison of Average, Peak, and Peak-to-Average Ratios in Force-Based Metrics

**DOI:** 10.3390/s25010151

**Published:** 2024-12-30

**Authors:** Gabriel J. Sanders, Stacie Skodinski, Corey A. Peacock

**Affiliations:** 1 College of Education, Criminal Justice and Human Services, University of Cincinnati, Cincinnati, OH 45221, USA; 2 College of Healthcare Sciences, Nova Southeastern University, Fort Lauderdale, FL 33328, USA; cpeacock@nova.edu

**Keywords:** wearable, data merging, predictors, force plate

## Abstract

The purpose was to create a systematic approach for analyzing data to improve predictive models for fatigue and neuromuscular performance in volleyball, with potential applications in other sports. The study aimed to assess whether average, peak, or peak-to-average ratios of countermovement jump (CMJ) force plate metrics exhibit stronger correlations and determine which metric most effectively predicts performance. Data were obtained from nine division I female volleyball athletes over a season, recording daily jump loads (total jumps, jump counts >38.1 cm (Jumps 38+), and >50.8 cm (Jumps 50+) in height) and comparing these with CMJ force metrics recorded the next day, both average and peak. Correlations and regressions were utilized to assess the relationship and predictive value for jump loads on CMJ test data. The findings revealed that the most significant (*p* < 0.001 for all) negative correlations (*r* ranged from −0.384 to −0.529) occurred between Jumps 50+ and the average CMJ test variables. Furthermore, there were no significant relationships between jump loads and peak-to-average ratios (*p* ≥ 0.233). Average CMJ force metrics and Jumps 50+ provide slightly more predictive (up to 28% of variability) potential for fatigue modeling of neuromuscular performance.

## 1. Introduction

Neuromuscular function plays a pivotal role in influencing both athletic performance and the likelihood of injury among elite volleyball players, primarily due to the increased jump demands [1,2,3,4]. Defined as a decline in the muscle’s ability to produce maximal force or power due to sustained physical activity, neuromuscular performance can be influenced by a range of physiological and neurological factors [1,2,5]. Excess fatigue from high jump loads reduces ankle contact angles and range of motion, impairs energy dissipation, and increases ACL forces, while also exacerbating knee valgus and internal rotation moments during single-leg landings, thereby heightening ACL injury risk [6,7]. Effective monitoring of this fatigue is essential, especially during high-intensity periods, such as the start of a competitive season and then throughout the season, where optimal recovery and readiness are vital to performance [2,8]. Countermovement jump (CMJ) tests are frequently used to assess neuromuscular status because they are non-fatiguing, provide insights into key performance metrics, such as force production, velocity, and power, which are integral to explosive movements in volleyball [9]. The test’s ease of use and ability to capture fatigue-related neuromuscular changes make it a preferred choice in both field and laboratory settings, where regular, reliable data can guide training adjustments and prevent injury [10,11]. For volleyball athletes, tracking fatigue through CMJ tests is especially relevant, as it can help optimize workload distribution and maintain peak performance levels throughout the season [5].

Force plates are widely recognized as the gold standard for analyzing CMJ performance, allowing for precise measurements of kinetic parameters such as peak force, rate of force development (RFD), and propulsive force [10]. However, variability in CMJ data can stem from multiple factors, including athlete technique, diurnal influences, and testing procedures, complicating the analysis and interpretation of results for fatigue assessment [12,13]. Studies suggest that peak performance metrics may best capture an athlete’s maximum capacity by reducing the influence of suboptimal attempts, yet this approach can miss variability caused by fatigue across multiple jumps [10,13,14]. Conversely, using average data values across multiple jumps can provide a more stable and reliable measure by mitigating the impact of single outliers, but this approach may lack sensitivity to acute neuromuscular performance [10,13].

In sports science, both peak and average CMJ values from any CMJ test metric (i.e., jump height, concentric and eccentric force, etc.) offer unique insights into athletic performance, and each has specific applications when assessing neuromuscular performance or training readiness. Peak performance metrics capture an athlete’s maximum capacity, providing a direct measure of power that is especially relevant in high-stakes or competitive scenarios where top output is critical [13]. Using peak values from any CMJ test metric can mitigate the influence of occasional suboptimal jumps due to temporary fatigue, poor technique, or psychological factors, thus offering a clearer view of the athlete’s true performance ceiling [12]. For example, Taylor et al. [13] observed that peak measurements in jump assessments often better reflect maximal capacity, as they minimize the impact of inconsistencies that may skew average values.

On the other hand, average values from multiple CMJ attempts are commonly used to enhance reliability, as they provide a more consistent representation of performance by balancing out any outliers [10]. Hopkins et al. [15] emphasized that averaging multiple attempts reduces variability and enhances stability in metrics, which is especially valuable when monitoring performance over time or tracking athlete fatigue. However, averaging can mask peak performance, as it incorporates both high and low values, potentially underestimating an athlete’s true capability during an optimal jump [16,17,18]. Cormack et al. [19] suggest that averaging repeated measures offers a stable indicator of performance, ideal for load monitoring and predicting overall readiness. Taylor et al. [13] discusses the variability in iso-inertial jump assessments, highlighting that peak measurements can better reflect an athlete’s maximal capacity by mitigating the impact of occasional suboptimal attempts, while average values provide a more stable representation of performance over time. To harness the strengths of both measures, combining peak and average values as independent predictors in a model can provide a nuanced view of an athlete’s capacity and consistency. Mathematically, calculating a “peak-to-average ratio” could further inform on performance variability, with ratios close to 1 indicating consistent output, while higher ratios might highlight variability due to fatigue or inconsistency. This comprehensive approach allows for a more robust assessment of athlete performance and readiness.

In high-performance sports, wearable technology is essential for monitoring athletes’ workloads, providing a clear link between daily activities (i.e., jump loads or internal training loads) and neuromuscular or fatigue assessments conducted afterward [8,16,20,21]. The purpose of the study was to determine which metric; average, peak, or the peak-to-average ratios of force-based metrics derived from two CMJ tests, most accurately predict changes in neuromuscular performance. Therefore, it is hypothesized that peak force-based metrics derived from CMJ tests will more accurately predict changes in neuromuscular performance following daily jump loads compared to average force metrics and peak-to-average ratios. Identifying the most precise indicators of performance decrements could broaden practitioners’ applicability beyond volleyball to other sports that rely heavily on jump metrics.

## 2. Materials and Methods

Data was collected from nine NCAA Division I female volleyball players (N = 9, ages 19.4 ± 1.3 years; height 184.0 ± 7.1 cm) over the course of a 16-week competitive division I collegiate volleyball season. Body weight was excluded from the analysis due to university restrictions on body composition data collection for athletes. All participants were medically cleared by the team physician to engage in training and competition. To be included in the study, athletes needed to participate fully in each practice session or game and remain injury-free the following morning in order to complete two CMJs on a dual force plate. Only CMJ tests recorded within 24 h of the previous day’s jump load data were used for the analysis. A total of 313 observations (i.e., days of jump load data corresponding to CMJ tests the following morning) met the inclusion criteria and were analyzed across the 16-week season. Daily jump loads were recorded using wearable technology, with corresponding CMJ assessments conducted the following morning. While athletes did provide informed consent for the use of their data, the data collection was part of the university’s standard strength and conditioning program and typical collegiate athletic activities. The study received approval from the university’s institutional review board.

### 2.1. Jump Load and CMJ Data Collection

Jump load monitoring was carried out using a validated inertial measurement unit, a compact sensor device featuring a 3-axis accelerometer, gyroscope, and magnetometer (VERT 3, Fort Lauderdale, FL, USA) [22,23,24]. The units were fastened at the top of the iliac crest using a tight band, following the manufacturer’s guidelines. Data from the wearable devices included total jump counts, jumps >38.1 cm (Jumps 38+), and jumps >50.8 cm (Jumps 50+), categorized by the manufacturer. Due to the indoor nature of volleyball, this GPS-free wearable device was utilized by each athlete during all practices and games throughout the season. To evaluate neuromuscular performance, CMJ tests were performed before any practice sessions utilizing a validated, portable dual force plate system (Hawkin Dynamics, Westbrook, ME, USA) with a sampling rate of 1000 Hz [25]. The CMJ tests were scheduled in the morning before any planned warm-up or additional training conducted by the strength coach, to prevent neural activation which could artificially enhance CMJ performance, potentially obscuring true neuromuscular capabilities [26].

During each successful CMJ test, athletes were instructed to remain still on the force plates with their hands on their hips to allow for proper system calibration. Upon the system’s readiness beep, athletes were prompted to execute a maximal effort CMJ, keeping their hands on their hips throughout the jump and landing on the force plates, one foot on each plate. Each test comprised two jumps, then the average (average value from two jumps), peak (best jump), and peak-to-average ratio (e.g., calculated as peak value/average value) of these attempts were subsequently analyzed. The force plate system captured and recorded data on variables such as force, velocity, and power throughout the jump and landing phases, which were later analyzed in connection with the daily jump load data.

### 2.2. Force-Based Metrics

Force metrics directly measure an athlete’s ability to generate and manage mechanical loads, which is fundamental for optimizing training and enhancing performance in power-dependent sports [27]. Jump height (centimeters, cm) refers to the vertical distance an athlete achieves from the ground during a jump, measured from the highest point reached relative to the takeoff position. Countermovement depth is the maximum depth (centimeters, cm) or the lowest point reached by an athlete during the countermovement phase of a jump before reversing direction to propel upwards. Force at minimum displacement is the force exerted at the lowest point of the athlete’s center of mass during the countermovement phase, which is crucial for reversing the downward motion and initiating upward propulsion.

Average braking force (Newtons, N) represents the average force applied during the braking phase of the jump, where the athlete decelerates and changes direction from moving downwards to moving upwards. Peak braking force (Newtons, N) is the highest force recorded during the braking phase and indicates the athlete’s ability to absorb and stabilize force effectively before the upward propulsion begins. Average propulsive force (Newtons, N) is the average force applied during the propulsive phase of the jump, where the athlete accelerates upwards, leaving the ground. Peak propulsive force (Newtons, N), on the other hand, is the maximum force exerted during the propulsive phase, indicating a stronger and more explosive jump.

Flight time (seconds) measures the duration the athlete’s feet are off the ground between the takeoff and landing phases of the jump, directly reflecting the airtime during a jump. The reactive strength index (RSI) is calculated as the flight time divided by ground contact time (GCT) (seconds) and is used to evaluate an athlete’s explosive leg strength and reactive abilities. Similarly, the modified reactive strength index (mRSI) is calculated as jump height divided by GCT, providing an alternative measure of an athlete’s reactive strength by focusing more on the effectiveness of the jump height relative to contact time. These metrics are essential in assessing an athlete’s neuromuscular capability and performance in sports requiring explosive movements.

### 2.3. Statistical Analysis

Descriptive statistics, including means and standard deviations, were calculated for all jump load metrics (total jump counts, Jumps 38+, and Jumps 50+) and for CMJ force-based metrics, including average, peak, and peak-to-average ratios for each metric. Normality of the data was assessed using the Shapiro-Wilk test to ensure appropriateness for parametric analysis.

To evaluate the initial relationships between jump loads and CMJ metrics, Pearson’s correlation coefficients were calculated for total jump counts, Jumps 38+, and Jumps 50+ with average, peak, and peak-to-average ratios of CMJ force-based metrics. Based on the correlation results, variables demonstrating the strongest relationships were further examined using simple linear regression analyses. Specifically, linear regression models were used to evaluate the predictive potential of Jumps 50+ on both average and peak CMJ test force metrics, as this category of jump loads showed stronger correlations with CMJ variables compared to total jump counts and Jumps 38+. Model assumptions, including linearity and homoscedasticity, were checked using Durbin–Watson statistics.

A post-hoc power analysis was conducted to evaluate the study’s ability to detect significant effects in the regression models. With a total of 313 observations, one predictor variable, and a significance level of α = 0.05, the analysis confirmed a statistical power of 0.80, which is sufficient for detecting meaningful relationships. The analysis ensures that the sample size was adequate to support the reliability of the findings, reinforcing the robustness of the study design while addressing the exploratory nature of the research.

The focused analysis on Jumps 50+ allowed for a targeted investigation into the direct relationship between high-intensity jump activities and neuromuscular performance, as quantified by CMJ test outcomes. The results provided insight into the effects of the most physiologically demanding jump loads on subsequent fatigue markers. Statistical significance was set a priori at *p* < 0.05. All statistical analyses were performed using IBM SPSS Statistics version 29.0.

## 3. Results

### 3.1. Descriptives for Jump Loads and CMJ Test

Descriptive statistics were calculated for total jump counts (88.5 ± 51.5), Jumps 38+ (49.7 ± 37.1) and Jumps 50+ (23.7 ± 28.5) and then for average, peak, and peak-to-average ratios for all CMJ test force-based metrics (Table 1).

### 3.2. Correlations for Jump Loads and CMJ Test Correlations

The correlations between jump loads and all force metrics are provided in Table 2. There were significant negative relationships between jump loads and average (Two CMJ test average) and peak (greatest CMJ test jump) force-based metrics, demonstrating that increases in the highest intensity jump loads were related to decreases in force-based metrics, not including reactive strength and modified reactive strength indices. Notably, Jumps 50+ was most strongly correlated to all variables with trivial variations between using average or peak CMJ test values. There were no significant correlations between jump loads of any intensity and peak-to-average ratios for any force plate metrics, thus the peak-to-average values may not have value when analyzing neuromuscular performance data from jump loads and CMJ tests.

### 3.3. Regressions for Jumps 50+ and Average and Peak CMJ Test

The results from the linear regression models assessing the impact of Jumps 50+ on average and peak CMJ force metrics reveal notable findings regarding the predictive utility of using either average or peak values to discern alterations in neuromuscular performance (Table 3). Further, several CMJ metrics including Braking RFD, Countermovement Depth, and various force measures showed statistically significant negative relationships with Jumps 50+, indicating that higher jump counts lead to decreased performance metrics. Both average and peak values similarly reflect these trends, with peak values showing slightly more sensitivity to high-intensity jumps. Average CMJ metrics may have a marginally better predictive value due to slightly higher R^2^ values.

## 4. Discussion

The primary findings revealed average CMJ force-based metrics, including braking RFD, countermovement depth, force at minimum displacement, average braking force, peak braking force, average propulsive force, and peak propulsive force, have statistically significant, negative relationships with Jumps 50+. This suggests greater counts of higher intensities jumps may adversely affect neuromuscular performance, as indicated by a decrease in these force metrics. Notably, the use of average CMJ values provides a slightly better predictive framework compared to peak values. This is reflected in marginally higher R^2^ values for average metrics, implying a more consistent and comprehensive representation of the data trends across the season.

Recent research supports the finding that peak-to-average ratios in countermovement jump (CMJ) metrics do not significantly enhance the prediction of neuromuscular performance. A study by Claudino et al. [11] found that while both peak and average CMJ metrics individually provide valuable insights into an athlete’s performance, their ratio does not offer additional predictive value, thus corresponding to our results. Further confirmed by the current results, focusing on average CMJ values may yield more reliable and actionable insights for monitoring and adjusting training loads to optimize athletic performance.

The findings this study align with and extend previous research that has examined the relationship between jump loads and CMJ metrics in monitoring neuromuscular performance [2,8,28,29]. Sanders et al. [28] identified significant relationships between jump loads and force-velocity metrics, highlighting that positional demands influence CMJ outcomes across a competitive volleyball season. Similarly, Cabarkapa et al. [2] observed season-long variations in CMJ metrics, with concentric measures such as mean force and power remaining stable, while eccentric metrics like peak velocity and power fluctuated, particularly during tapering phases. These results mirror the current study’s observation that average CMJ force-based metrics, such as braking force and propulsive force, provide a more consistent and reliable indication of workload-induced neuromuscular changes compared to peak or peak-to-average ratios.

Gathercole et al. [29] further emphasized the utility of CMJ metrics in detecting acute neuromuscular fatigue, noting that variables like peak power and force at zero velocity were sensitive to fatigue-induced changes immediately following exercise. These previous findings align with the current study’s evidence that high-intensity jump loads negatively impact average CMJ metrics, reinforcing the concept that fatigue is more effectively captured through average values. Unlike isolated peak values, which may reflect momentary performance, average metrics provide a comprehensive representation of aggregate trends across the season. This approach offers more actionable insights for optimizing training and recovery strategies in real-world competitive settings. Together, these studies underscore the value of CMJ tests as a non-invasive, practical tool for monitoring fatigue and performance decrements in high-performance sports.

Likewise, integrating additional data points such as biometric indicators (e.g., heart rate variability, cortisol levels), comprehensive training load data, and nutritional intake can enhance predictive models. For instance, a study by McGuigan et al. [30] demonstrated that combining CMJ metrics with heart rate variability and training load data improved the accuracy of performance predictions. For instance, interaction terms between Jumps 50+ and recovery metrics or polynomial features to capture non-linear effects could be developed to enhance predictive accuracy. Advanced machine learning (ML) techniques, such as Random Forests, Gradient Boosting Machines, and Neural Networks, are particularly well-suited for these tasks due to their robustness against overfitting and ability to model complex, non-linear relationships [31]. As demonstrated by Morciano et al. [32], Random Forest models trained on specific combinations of input parameters can achieve predictive accuracies exceeding 90% across distinct athlete roles when optimized. This highlights the potential of role-specific training and forecasting models in sports.

Operationalizing these models within sports science or athlete management systems allows for real-time, dynamic assessments, facilitating ongoing adjustments to training based on predictive insights [31,32]. For example, models can forecast when an athlete approaches a fatigue threshold, enabling proactive modifications to training loads and mitigating injury risks. Embedding these predictive tools into decision-making frameworks offers sports organizations the ability to design customized training regimens, enhance tactical decision-making, and guide recruitment strategies. Employing robust cross-validation techniques and frequent model updates ensures that predictions remain accurate and reflective of an athlete’s evolving state, further aligning with findings that role-specific modeling enhances precision. These advanced tools empower sports teams to optimize performance while minimizing the likelihood of injury, creating a data-driven foundation for athlete development and management [33].

The correlation and regression analyses serve as a foundational step in identifying key relationships between high intensity jump counts, like Jumps 50+, and various CMJ metrics, providing critical insights for more sophisticated machine learning models [34]. In sports science research, the integration of artificial intelligence (AI) and ML technologies is pivotal for efficiently managing complex datasets, especially when coordinating and aligning data across variables such as jump loads and CMJ test outcomes [34]. AI facilitates the automation of data merging and alignment processes, ensuring that data entries are precisely matched based on key identifiers like dates and athlete names, a critical step for accurate longitudinal analyses [35]. For instance, ensuring that jump loads from one session correctly correspond to CMJ test performance on subsequent days is essential for valid causal inference [8]. AI tools streamline this process by employing advanced date-time parsing and synchronization techniques, significantly reducing human error and increasing the reliability of the dataset for statistical analyses. Utilizing an ’inner merge’ approach, AI systems can ensure that only records with complete data from both datasets are included in the analysis, thus preventing the inclusion of incomplete records that could potentially skew the results [35]. By leveraging data manipulation libraries such as Python’s Pandas researchers are equipped to handle these processes with greater accuracy and efficiency [36]. This methodological rigor supports the scientific validity of sports research, facilitating more informed decisions about training programs and athlete performance management.

While this study is the first to link any type of workload to CMJ test results across an entire season, it is not without limitations. The workload and neuromuscular performance data is confined to a single sports team and is also not differentiated by position, which may limit the generalizability of the findings across different positions and athletic populations [17,18]. Various teams or different sports require vastly different physical demands, and training is widely different and could exhibit distinct physiological responses to similar training loads. Additionally, the study relies solely on two measurement types: wearable technology for recording jump loads and force plates for conducting CMJ tests. This approach may overlook other influential factors such as psychological stress, muscle fatigue, or external environmental conditions, which are not captured by the current measurement tools.

Looking ahead, advancements in wearable technology could enhance the granularity of data collection by categorizing jump load intensities into specific height ranges (0–20 cm, 20–40 cm, and 40+ cm) and capturing changes in direction and additional movement dynamics [37]. Enhanced data collection from additional measures would offer deeper insights into the physical demands placed on athletes during training and competition and AI and ML would be able to digest the robust increase in metrics. Future research could leverage these advanced data collection techniques for more comprehensive models that encompass a broader spectrum of variables influencing athlete performance. Integrating these technologies seamlessly, either in the weight room or alongside other measurement tools, could transform athletic training into a more data-driven and precise science, enhancing both performance and injury prevention.

## 5. Conclusions

The results indicate that Jumps 50+ and average CMJ force metrics provide a slightly more accurate model for assessing neuromuscular performance in collegiate volleyball athletes during the season compared to peak force-based metrics, total jump counts, and Jumps 38+. Although the improvement of the average CMJ model over the peak model is subtle, in elite sports, where the difference between winning and losing is often minimal, this small enhancement in data precision can be critical. Consequently, average force metrics from CMJ tests appear to be more reliable indicators for monitoring athlete fatigue and neuromuscular performance. These findings hold significant potential for developing advanced prediction models using machine learning and AI. By incorporating average force metrics along with data on high-intensity movements like Jumps 50+, such models could enable coaches and sports scientists to better predict fatigue, optimize performance, and prevent injuries. Leveraging AI allows for the refinement of training protocols and enhances athlete care through precise, data-driven decision-making.

## Figures and Tables

**Table 1:** Descriptive statistics for CMJ test metrics. Data are means ± SD.

	Average	Peak	Peak-to-Average Ratios
Jump Height (cm)	31.5 ± 5.8	32.3 ± 6.4	1.025 ± 0.026
Flight Time (seconds)	0.539 ± 0.048	0.543 ± 0.049	1.007 ± 0.015
Braking RFD (N/s)	5216.7 ± 2202.1	5350.7 ± 2332.2	1.026 ± 0.097
Countermovement Depth (cm)	−38.8 ± 5.4	−38.8 ± 5.6	0.999 ± 0.040
Force at Min Displacement (N)	1690.7 ± 275.8	1704.4 ± 282.0	1.008 ± 0.023
Average Braking Force (N)	1391.6 ± 237.5	1400.9 ± 243.3	1.007 ± 0.015
Peak Braking Force (N)	1720.1 ± 296.6	1734.5 ± 302.5	1.008 ± 0.012
Average Propulsive Force (N)	1321.9 ± 170.8	1328.8 ± 172.1	1.005 ± 0.021
Peak Propulsive Force (N)	1716.2 ± 251.4	1727.3 ± 258.6	1.006 ± 0.012
Reactive Strength Index (RSI)	0.63 ± 0.07	0.64 ± 0.08	1.010 ± 0.060
Modified-RSI (mRSI)	0.37 ± 0.07	0.38 ± 0.08	1.025 ± 0.067

**Table 2:** Correlation results for the relationship between jump loads and average, peak, and peak-to-average ratios for each CMJ force-based metric.

Total Jump Counts	Average Values		Peak Values		Peak-to-Average	
	Pearson’s *r*	*p*-Value	Pearson’s *r*	*p*-Value	Pearson’s *r*	*p*-Value
Jump Height (cm)	−0.153	0.007	−0.142	0.012	0.031	0.586
Flight Time (seconds)	−0.178	0.002	−0.179	0.002	−0.003	0.956
Braking RFD (N/s)	0.189	<0.001	0.168	0.003	−0.055	0.331
Countermovement Depth (cm)	0.128	0.023	0.130	0.022	−0.023	0.688
Force at Min Displacement (N)	0.189	<0.001	0.171	0.002	−0.068	0.233
Average Braking Force (N)	0.224	<0.001	0.209	<0.001	−0.049	0.391
Peak Braking Force (N)	0.199	<0.001	0.177	0.002	−0.052	0.361
Average Propulsive Force (N)	0.125	0.026	0.121	0.033	−0.044	0.440
Peak Propulsive Force (N)	0.210	<0.001	0.190	<0.001	−0.061	0.285
Reactive Strength Index (RSI)	−0.009	0.876	0.005	0.924	0.037	0.510
Modified-RSI (mRSI)	−0.084	0.140	−0.062	0.276	0.051	0.369
**Jumps 38+**	**Average Values**		**Peak Values**		**Peak-to-Average**	
	**Pearson’s *r***	***p*-Value**	**Pearson’s *r***	***p*-Value**	**Pearson’s *r***	***p*-Value**
Jump Height (cm)	0.204	<0.001	0.201	<0.001	0.014	0.801
Flight Time (seconds)	0.251	<0.001	0.245	<0.001	−0.022	0.703
Braking RFD (N/s)	−0.333	<0.001	−0.331	<0.001	0.000	0.993
Countermovement Depth (cm)	−0.266	<0.001	−0.259	<0.001	0.010	0.860
Force at Min Displacement (N)	−0.359	<0.001	−0.360	<0.001	0.000	0.996
Average Braking Force (N)	−0.373	<0.001	−0.370	<0.001	−0.008	0.895
Peak Braking Force (N)	−0.362	<0.001	−0.369	<0.001	−0.044	0.439
Average Propulsive Force (N)	−0.299	<0.001	−0.304	<0.001	−0.025	0.666
Peak Propulsive Force (N)	−0.334	<0.001	−0.338	<0.001	−0.017	0.763
Reactive Strength Index (RSI)	0.021	0.706	−0.002	0.972	−0.048	0.401
Modified-RSI (mRSI)	0.098	0.083	0.084	0.137	−0.030	0.595
**Jumps 50+**	**Average Values**		**Peak Values**		**Peak-to-Average**	
	**Pearson’s *r***	***p*-Value**	**Pearson’s *r***	***p*-Value**	**Pearson’s *r***	***p*-Value**
Jump Height (cm)	0.497	<0.001	0.487	<0.001	0.021	0.716
Flight Time (seconds)	0.499	<0.001	0.484	<0.001	−0.061	0.281
Braking RFD (N/s)	−0.419	<0.001	−0.411	<0.001	0.012	0.837
Countermovement Depth (cm)	−0.384	<0.001	−0.350	<0.001	−0.051	0.364
Force at Min Displacement (N)	−0.529	<0.001	−0.526	<0.001	−0.023	0.682
Average Braking Force (N)	−0.526	<0.001	−0.520	<0.001	−0.018	0.752
Peak Braking Force (N)	−0.529	<0.001	−0.528	<0.001	−0.010	0.857
Average Propulsive Force (N)	−0.416	<0.001	−0.418	<0.001	−0.003	0.961
Peak Propulsive Force (N)	−0.480	<0.001	−0.478	<0.001	−0.006	0.912
Reactive Strength Index (RSI)	0.219	<0.001	0.171	0.002	−0.037	0.520
Modified-RSI (mRSI)	0.382	<0.001	0.351	<0.001	−0.008	0.887

**Table 3:** Linear regression models for Jumps 50+ on average and peak CMJ force-based variables.

	Average Values					Peak Values				
CMJ Variable	Constant	β	*p*-Value	R^2^	RMSE	Constant	β	*p*-Value	R^2^	RMSE
Braking RFD (N/s)	5982.8	−32.372	<0.001	0.176	2002.8	6146.4	−33.620	<0.001	0.169	2129.8
Countermovement Depth (cm)	−37.1	−0.073	<0.001	0.147	5.0	37.2	−0.069	<0.001	0.123	5.2
Force at Min Displacement (N)	1812.0	−5.125	<0.001	0.280	234.4	1827.6	−5.203	<0.001	0.277	240.3
Average Braking Force (N)	1495.3	−4.381	<0.001	0.277	202.4	1506.0	−4.438	<0.001	0.270	208.1
Peak Braking Force (N)	1850.3	−5.503	<0.001	0.280	252.2	1867.3	−5.609	<0.001	0.279	257.2
Average Propulsive Force (N)	1380.8	−2.491	<0.001	0.173	155.6	1388.5	−2.523	<0.001	0.175	156.6
Peak Propulsive Force (N)	1816.4	−4.231	<0.001	0.230	220.9	1830.1	−4.343	<0.001	0.228	227.5

## Data Availability

Due to university restrictions, the data supporting the findings of this study are not publicly available. Requests for data access should be directed to the corresponding author, G.S., who will process them in accordance with the policies of the university and applicable legal requirements.
